# Genome-wide association study reveals candidate loci for resistance to anthracnose in blueberry

**DOI:** 10.1093/g3journal/jkaf187

**Published:** 2025-09-08

**Authors:** Lushan Ghimire, Paul Adunola, Philip F Harmon, Camila F Azevedo, Tyler J Schultz, Bruno Leme, Felix Enciso-Rodriguez, Juliana Benevenuto, Luis Felipe V Ferrão, Patricio R Munoz

**Affiliations:** Blueberry Breeding and Genomics Lab, Horticultural Sciences Department, University of Florida, Gainesville, FL 32611, United States; Blueberry Breeding and Genomics Lab, Horticultural Sciences Department, University of Florida, Gainesville, FL 32611, United States; Plant Pathology Department, University of Florida, Gainesville, FL 32611, United States; Blueberry Breeding and Genomics Lab, Horticultural Sciences Department, University of Florida, Gainesville, FL 32611, United States; Department of Statistics, Universidade Federal de Viçosa, Viçosa, MG 36570-000, Brazil; Blueberry Breeding and Genomics Lab, Horticultural Sciences Department, University of Florida, Gainesville, FL 32611, United States; Blueberry Breeding and Genomics Lab, Horticultural Sciences Department, University of Florida, Gainesville, FL 32611, United States; Blueberry Breeding and Genomics Lab, Horticultural Sciences Department, University of Florida, Gainesville, FL 32611, United States; Blueberry Breeding and Genomics Lab, Horticultural Sciences Department, University of Florida, Gainesville, FL 32611, United States; Blueberry Breeding and Genomics Lab, Horticultural Sciences Department, University of Florida, Gainesville, FL 32611, United States; Blueberry Breeding and Genomics Lab, Horticultural Sciences Department, University of Florida, Gainesville, FL 32611, United States; Plant Breeding Graduate Program, University of Florida, Gainesville, FL 32611, United States

**Keywords:** *Vaccinium*, breeding, genomics, disease resistance, *Colletotrichum gloeosporioides;* plant genetics and genomics

## Abstract

Anthracnose, caused by *Colletotrichum gloeosporioides*, poses a significant threat to blueberries, necessitating a deeper understanding of the genetic mechanisms underlying resistance to develop efficient breeding strategies. Here, we conducted a genome-wide association study on 355 advanced selections of southern highbush blueberry from the University of Florida Blueberry Breeding and Genomics Program. Visual scores and image analyses were used for assessing disease severity. The population was genotyped using Capture-Seq, detecting 38,379 single nucleotide polymorphisms. The study revealed a moderate narrow-sense heritability estimate (∼0.5) for anthracnose resistance in blueberries. Minor additive loci contributing to anthracnose resistance were identified on chromosomes 2, 3, 5, 6, 9, 10, and 12, using 2 different phenotyping approaches. Visual and image-based phenotyping captured complementary aspects of anthracnose resistance, identifying distinct, non-overlapping SNP associations. Candidate gene mining flanking significant associations unveiled key defense-related proteins, such as serine/threonine protein kinases, pentatricopeptide repeat-containing proteins, E3 ubiquitin ligases that have been well-known for their roles in plant defense signaling pathways. Our findings highlight the complex and quantitative resistance mechanism for anthracnose in blueberry, providing insights for breeding strategies and sustainable disease management.

## Introduction

Anthracnose is a detrimental fungal disease of significant global importance, impacting a broad range of crops, including several high-value horticultural commodities such as strawberries, grapes, raspberries, blackberries and blueberries. In blueberries, the disease can compromise overall plant health and vigor, and lead to substantial pre- and postharvest losses due to fruit rot and reduced shelf life. The *Colletotrichum* genus, causing this disease, ranks among the top plant pathogen genera globally for its economic impact (Dean et al. 2012). Within it, *Colletotrichum gloeosporioides* is reported to be the main cause of anthracnose in southern highbush blueberries (SHB; *Vaccinium corymbosum* and hybrids) (Velez-Climent and Harmon 2016; Phillips et al. 2018a). It has also been reported to affect blueberries worldwide, in regions that include North America, Japan, Spain, South Korea, and China (Barrau et al. 2001; Yoshida and Tsukiboshi 2002; Polashock et al. 2005; Yu et al. 2005; Kim et al. 2009). Anthracnose-induced yield loss in blueberries has been documented to exceed 50% by the third harvesting cycle in New Jersey and under poor storage conditions, losses of up to 100% are possible (Polashock et al. 2017)

Anthracnose symptoms include sunken lesions with conidial masses, leading to substantial dieback, affecting leaf, stem and fruit tissues, diminishing production, and rendering fruits unmarketable (Phillips et al. 2018a). Conidia germinate and can initiate infection through direct penetration via appressoria or through wounds and natural openings (Jeffries et al. 1990). No commercial blueberry cultivars are immune to anthracnose; thus, fungicides remain the primary strategy for disease management. Among these, quinone outside inhibitor (QoI) fungicides are commonly used; they inhibit fungal respiration by binding to the quinol-oxidation site of the cytochrome bc_1_ complex, blocking electron transfer and halting ATP production (Fernández-Ortuño et al. 2008). However, resistance to QoI fungicides has already been reported in blueberry farms in Florida (Harmon 2014), underscoring the need for alternative control methods.

Leveraging genetic sources of disease resistance presents a more sustainable and cost-effective alternative to chemical management (Polashock et al. 2005). However, improving host resistance through traditional breeding is time-consuming and resource intensive (Slater et al. 2013). Recent advancements in sequencing and automation technologies have substantially lowered genotyping costs, facilitating the identification of molecular markers associated with desirable traits. Modern breeding strategies have successfully developed disease resistance to anthracnose in several plant species such as grapes (Jang et al. 2020), pepper (Suwor et al. 2017), sorghum, (Ramasamy et al. 2009) and mango (Felipe et al. 2022). In contrast, blueberry breeding has relied primarily on phenotypic selection, with limited deployment of molecular tools for disease resistance. While resistant germplasm has been identified for pathogens including *Ralstonia solanacearum, Botryosphaeria dothidea, Phomopsis vaccinii, Colletotrichum acutatum* (Polashock et al. 2005; Polashock and Kramer 2006; Conner et al. 2022), these resources have not been widely integrated into genomics-enabled breeding pipelines. Related small fruits such as cranberry and raspberry have made greater strides, with characterized genetic resources for disease resistance utilized in breeding programs (Ward et al. 2011; Johnson-Cicalese et al. 2015). Notably, genome-wide association studies (GWAS) in blueberries have been successfully implemented to dissect the genetic basis of key horticultural traits, such as fruit quality, off-season flowering, and flavor-related volatiles (Ferrão et al. 2018, 2020; da Silva et al. 2024), providing a foundation for extending this approach to disease resistance.

Despite the economic importance of anthracnose in blueberries, the genetic basis of this disease resistance remains poorly understood. Most prior studies have focused on anthracnose fruit rot during postharvest, caused by *Colletotrichum fioriniae* (formerly *Colletotrichum acutatum*) primarily in northern highbush blueberry (NHB) cultivars (Wharton and Schilder 2008; Miles et al. 2011; Miles and Hancock 2022; Jacobs et al. 2023). In the case of SHB in Florida, *C. gloeosporioides* is the predominant causal agent of anthracnose, affecting leaf, stem and fruit tissues (Velez-Climent and Harmon 2016; Phillips et al. 2018a, 2018b). While resistance in fruit tissues was not correlated with foliar resistance for *C. acutatum* (Ehlenfeldt et al. 2006), such relationships remain unexplored for *C. gloeosporioides*, which poses a greater concern for SHB. Notably, isolates from 1 tissue type have been reported to infect others (Phillips et al. 2018b). This distinction is particularly relevant in Florida's evergreen production system, where infected foliage and stems persist year-round and serve as recurring sources of inoculum.

To address this gap, we evaluated the genetic architecture of anthracnose resistance in SHB leaf and stem tissues inoculated with *C. gloeosporioides* using both visual and image-based assessments. To our knowledge, this is the first study to investigate the genetic basis of resistance to *C. gloeosporioides* in non-fruit tissues of SHB. By exploring the phenotypic and genetic variability across the studied SHB breeding population, we identified marker-trait associations and potential candidate genes underlying resistance. These findings provide deeper insights into tissue-specific resistance and support the development of more effective breeding strategies for anthracnose management in Florida's SHB production systems.

## Materials and methods

### Plant material

Phenotypic evaluations were performed in a total of 355 SHB genotypes from the University of Florida Blueberry Breeding program. The genotypes evaluated in this study originated from 2 sets of advanced clonal selections, managed under commercial conditions in Waldo, FL, USA. All individuals originated from a common breeding population and exhibit varying degrees of relatedness based on shared pedigree structure.

To efficiently manage experimental logistics while expanding genetic representation for association mapping, phenotypic evaluations were conducted in 2 independent batches, each representing a distinct screening event, evaluated at a specific time point. These screening batches are hereafter designated as Batch-I and Batch-II, respectively. Batch-I comprised 157 genotypes and was evaluated in May 2023, during the peak of postspring vegetative flush while Batch-II comprised 198 genotypes and was assessed in August 2023, following the emergence of new shoots after postharvest hedging. To facilitate data integration across screening batches and to account for potential variation between screenings, 28 genotypes from Batch-I were included as internal checks in Batch-II. For each genotype in both batches, 6 softwood cuttings approximately 40 cm in length were collected at the phenological stage considered optimal for propagation.

### Source of isolate and preparation of inoculum

A monoconidial isolate of *C. gloeosporioides* (isolate “15-646”) that was originally collected in 2015 from naturally infected stems of the “Flicker” cultivar on a commercial blueberry farm in Central Florida was used to prepare inoculum for both screening batches. This same isolate was previously characterized and validated for consistent pathogenicity and symptom development on SHB (Phillips et al. 2018a). The isolate was cultured on potato dextrose agar (PDA) incubated under continuous fluorescent lighting at 25 °C for 5 d, using inoculum freshly revived from long-term filter paper storage at 4 °C. Conidia were quantified utilizing a hemacytometer (Bright-Line Hemacytometer; Hausser Scientific, Horsham, PA, USA) and the concentration of the suspension was adjusted to 1 × 10^7^ conidia mL^−1^ with autoclaved distilled water for spray application. The conidia suspension was diluted to 1 × 10^4^ conidia mL^−1^ for dip inoculations. Control plants were sprayed with equal volumes of distilled water.

### Inoculation procedure

Two softwood cuttings per genotype were randomly chosen and bundled for each replication (replicate-bundles), with a total of 3 replications per genotype each with a unique QR-code. These replicate-bundles were wounded on the lower 1/3rd of the stem and placed over 3 144.8 cm × 45.7 cm × 19.7 cm plastic troughs (Model: G5435768, Bayhead Products, Zoro; USA). Each trough represented a randomized complete block with 1 replicate (2 cuttings) of each genotype. This entire experimental arrangement was housed within a self-constructed plastic tent situated in a growth room, consistently maintained at 90% to 95% relative humidity (Cool mist humidifier, Mikkin, USA) and 23 °C temperature (Supplementary Fig. 1a).

All the replicates were sprayed with the 1 × 10^7^ conidia mL^−1^ suspension until runoff, utilizing an aerosol spray gun (Crown Spra-Tool, Aervoe, NV, USA), thereby coating all stem surfaces and upper leaf surfaces. Each of the 3 troughs were filled with 40 L of the 1 × 10^4^ conidia mL^−1^ suspension. The genotypes were monitored daily for 35 d after inoculation. Following the 35-d incubation period, infection by *C. gloeosporioides* was confirmed through isolations from necrotic lesion boundaries excised from 5 random genotypes selected from each block. Symptomatic tissue was surface sterilized with a 10% bleach solution for 1 min, followed by three 1-min water rinses. Tissue was plated onto PDA and incubated under continuous fluorescent light at 25 °C until conidia with characteristic morphologies consistent with the original reference isolate were observed.

### Disease phenotyping

**Fig. 1.**
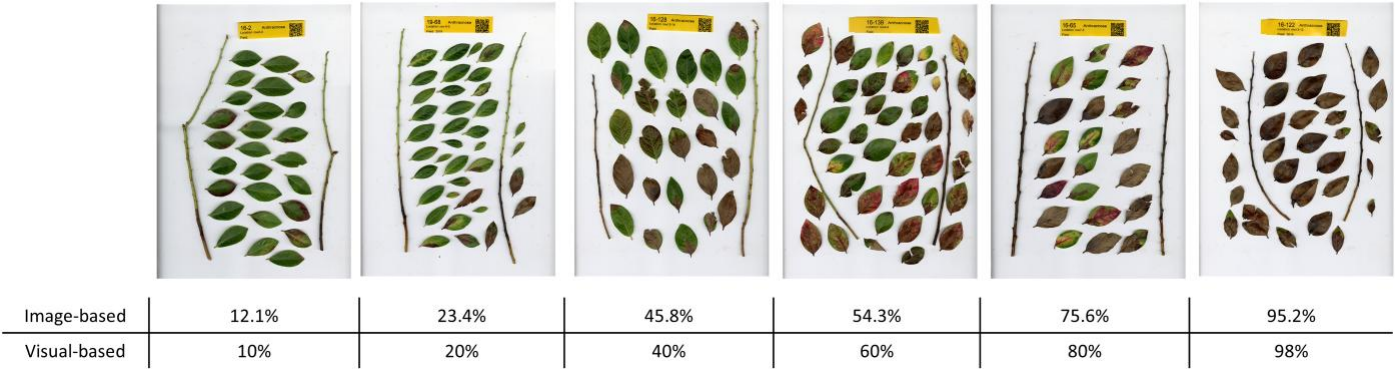
Disease severity scores (percentage) of different SHB genotypes in a continuous scale in response to anthracnose using the 2 different phenotyping approaches: image- and visual-based approaches.

In our study, we adopted 2 phenotyping approaches for disease quantification on day 35 postinoculation: visual inspection of the individuals by human eyes (visual-based) and the use of a custom-made automatic computer vision phenotyping method (image-based). Visual evaluation involved scoring disease severity on a continuous scale, as a percentage of the total area covered by necrotic leaf and stem lesions combined, as observed in natural field conditions. This score was recorded for each genotype within a replicate as an average value for the 2 stem cuttings per replicate. Simultaneously, comprehensive images encompassing all leaves and stem tissues for each genotype were captured, incorporating a unique QR-code (Fig. 1). Subsequently, these images underwent disease assessment through the computer vision phenotyping approach. The computer vision analysis used a naïve Bayesian machine learning methodology (Supplementary Fig. 1b, c) (Abbasi et al. 2016) to classify image pixels into 3 feature classes: healthy tissue, diseased tissue, and background. To train the model, we manually selected representative pixels from each class using 3 × 3-pixel grids sampled from 30 images. Model training and image analysis were performed using PlantCV version 3.13.0. (Gehan et al. 2017). These images were chosen such that 3 came from each 10% range of visually scored disease severity, covering the entire spectrum from 0% to 100%. This stratified sampling ensured that the pixel data used for training captured the full variability present in the dataset. The selected pixels were used to construct a multi-dimensional probability density function in the HSV (Hue, Saturation, Value) colorspace, capturing the color characteristics of each class. Once trained, the model was applied to segment each image by assigning individual pixels to their most probable class based on the learned color distributions. The disease severity (%) was then calculated from the segmentation masks using the formula:


$$\begin{array}{rl} \mathrm{Disease}\;\;\mathrm{severity}\left(\%\right)\;= & \;\mathrm{Diseased}\;\;\mathrm{area}/(\mathrm{Healthy}\;\;\mathrm{area}\\ & +\mathrm{Diseased}\;\;\mathrm{area})\times 100 \end{array}$$

### Genotypic data

Fresh, juvenile leaves were harvested from each genotype for subsequent genotyping procedures. DNA extraction and genotyping via Capture-Seq were performed at RAPiD Genomics (Gainesville, FL, USA). A genome-wide panel of 10,000 biotinylated probes, each spanning around 120-mers, were used for targeted capture, followed by high-throughput sequencing on the Illumina HiSeq2000 platform through 150-cycle paired-end runs.

SNP calling procedures adhered to the methodology by Ferrão et al. (2021). Raw reads underwent quality-based filtering and trimming. The filtered reads were aligned against the largest haploid scaffold set derived from the *V. corymbosum* cv. “Draper” genome (Colle et al. 2019) using Mosaik v.2.2.3 (Lee et al. 2014). SNPs were subsequently called with the 10,000 probe positions as targets employing FreeBayes v.1.3.2 (Garrison and Marth 2012). SNP filtering criteria included a minimum mapping quality of 10, exclusivity to biallelic loci, a maximum missing data of 50% and a minor allele frequency threshold of 0.01. We used Vcftools v. 0.1.16 to extract sequencing read counts per allele and individuals from the variant call file (Danecek et al. 2011).

The updog package v.2.1.0 in R was employed to ascertain allele dosages based on the read counts (Gerard et al. 2018). SNPs accurately genotyped in 95% of the individuals were retained (Supplementary File 1). The ploidy parameterization was encoded by the dosage of the alternative allele (B) in relation to the reference allele (A) as follows: 0 (AAAA), 1 (AAAB), 2 (AABB), 3 (ABBB), and 4 (BBBB) (Rosyara et al. 2016).

### Phenotypic analysis

**Fig. 2.**
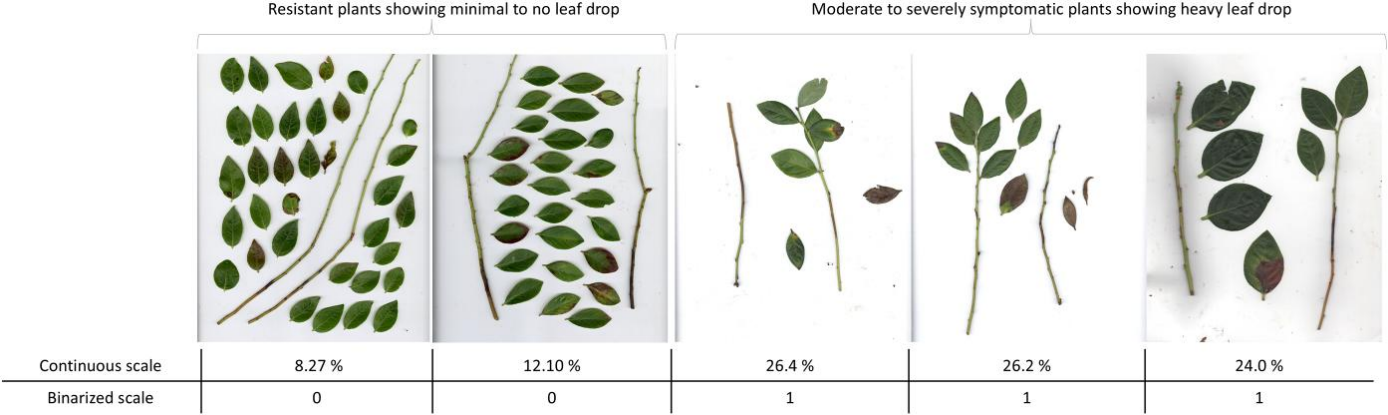
Comparative visualization of leaf drop in highly resistant vs moderately to severely symptomatic genotypes, showcasing disease severity scores on a continuous scale alongside their binary categorization.

We calculated the average of all 3 replicates within each batch measured on the continuous scale (Supplementary File 2). Also, in our study, we observed that some genotypes with moderate to severe anthracnose symptoms exhibited defoliation during incubation. In contrast, genotypes with lower symptom levels didn't exhibit visible leaf drop (Fig. 2). This observation raised concerns regarding the accuracy of severity assessment, particularly for genotypes falling within the moderate to severely symptomatic spectrum. To mitigate potential inaccuracies and improve precision in our analysis, we opted to simplify our dataset using a binary approach. We applied a stringent threshold of the 5th percentile on the respective batch's average severity across 3 replicates. This categorization involved assigning highly resistant genotypes with no to minimal leaf drop to category 0, while grouping genotypes exhibiting moderate to severe symptoms, with moderate to high leaf drop incidence into category 1. By adopting this classification method, we aimed to reduce the likelihood of errors in severity assessment and focus on discerning the extremes ends of resistance phenotypes. For the analysis, in each of the scales (continuous and binary), the distinct datasets obtained from both the screening batches were combined (Comb_popn), and a 1-stage correction was applied. This correction used the overlapping 28 genotypes from both batches as checks to correct for phenotyping batch effects and estimate the best linear unbiased estimates (BLUEs). Thus, adjusted means for each genotype were obtained using the following linear model:


(1) $$l_{ij}=\mu +B_{i}+G_{ij}+e_{ij}$$

where $l_{ij}$ is the liability for the phenotypic values represented by $y_{ij}$, $y_{ij}$ is the phenotype of genotype *i* in screening batch *j*; and they are associated by the identity link function for the continuous scale and logit link function for binary scale; *μ* is the overall mean; $B_{i}$ is the fixed batch effect; $G_{ij}$ is the genotypic effect of individual *i* at batch *j*; and $e_{ij}$ is the random residual effect. For the genotypic effect, the experiment was designed as an Augmented Block Design with common checks connecting different batches. Thus, the genotypic effect was separated into 2 groups, where *g* is the fixed effect for the regular individuals and *c* is the fixed effect associated with the checks. The BLUEs of each genotype were used as the response variable in subsequent analyses.

Furthermore, heritability estimates were computed to quantify the proportion of phenotypic variation attributed to genetic factors by considering genotypes as random effects and incorporating pedigree information into the relationship matrix:


(2) $$y^{*}=\;1\mu \;+\;Za\;+\;e\;,$$

where $y^{*}$ is the vector of BLUEs; *μ* represents the population mean with assumed flat prior distribution; e is the vector of random errors with $e∼N(O,I\sigma_{e}^{2})$ and $\sigma_{e}^{2}$ is the residual variance; and a is the vector of genomic estimated breeding values and its incidence matrix Z, with $a∼N(O,A\sigma_{a}^{2}),$ and $\sigma_{a}^{2}$ is the genetic variance. For variance components, we assumed a scaled inverted *χ*² distribution. The construction of the pedigree relationship matrix (A) was done using the AGHmatrix package in R (Amadeu et al. 2016). This model was implemented by the BGLR package in the R software (Pérez and de los Campos 2014), defining 500,000 iterations for the Markov chain Monte Carlo (MCMC) algorithms, a burn-in period of 50,000 MCMC cycles, and thin equals to 10 before saving samples from each, totaling 45,000 MCMC cycles. The heritability ($h^{2}$) was computed as the posterior mean: $h^{2}=\frac{\sigma_{a}^{2}}{\sigma_{a}^{2}+\sigma_{e}^{2}}\;$.

### Genome-wide association study

To identify the genomic regions regulating anthracnose resistance, GWAS was conducted using the R package GWASpoly v.2.11, specifically tailored for autopolyploid (Rosyara et al. 2016). For correction of population structure, the Q matrix was constructed using the top 5 principal components. Kinship matrices (K) were calculated using the algorithm embedded within the GWASpoly package. The testing of associations between SNPs and phenotypic variations was computed by the application of the Q + K linear mixed model (Rosyara et al. 2016) (Supplementary Files 3 and 4). An additive genetic model where the SNP effect scaled proportionally to allele dosage was explored. Adjusted Bonferroni correction, considering a significance level of 0.05, was used for establishing a *P*-value detection threshold. Associations between SNPs and phenotypes were examined using quantile-quantile plots of estimated −log10(*P*) values and linkage disequilibrium decay was visualized using the LD.plot function.

### Candidate gene mining

For the exploration of potential candidate genes post-GWAS, we selected genomic regions within a 100 kb window upstream and downstream of significantly associated SNPs using the “Draper” reference genome. The corresponding protein sequences were used as queries for comprehensive functional annotation using PANNZER (Törönen and Holm 2022) and eggNOG mapper (Huerta-Cepas et al. 2019) (Supplementary File 5). Candidate genes with functional description and gene ontology functions related to plant resistance or defense were selected and their potential functions were verified in literature search.

## Results

### Phenotypic analysis

**Fig. 3.**
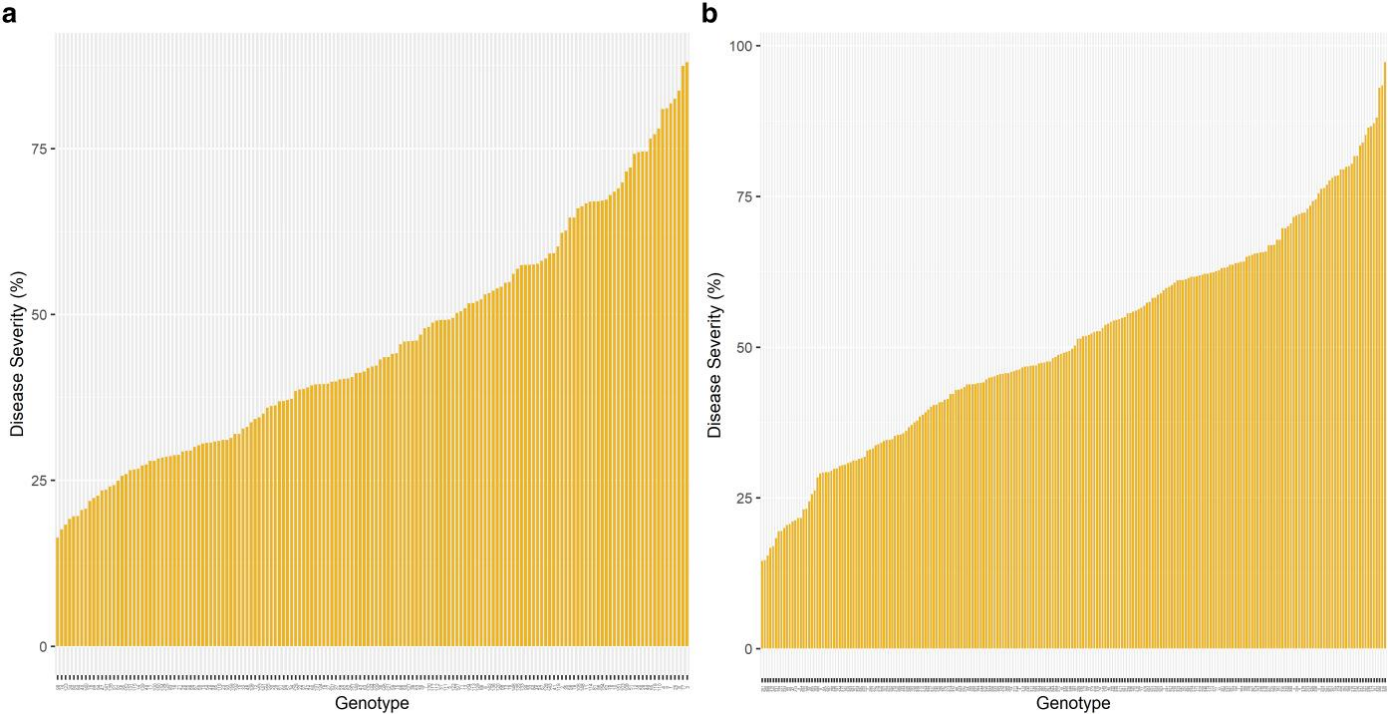
Susceptibility of southern highbush blueberry genotypes to anthracnose across 2 screening batches a) Batch-I and b) Batch-II using continuous dataset with image-based phenotyping approach. Each bar represents 1 genotype. The genotypes on the left-most end represent the most resistant genotypes while on the right-most end represent the most susceptible genotypes.

Image- and visual-based assessments of disease severity after *C. gloeosporioides* inoculation were performed in 2 screening batches (Batch-I and Batch-II). A significant correlation was observed between the 2 phenotyping approaches, image- and visual-based, in both screening batches, with values of 0.91 and 0.94 within Batch-I and Batch-II, respectively (Fig. 1; Supplementary Fig. 2). Broad phenotypic variation in disease severity was noted across both the screening batches. In accordance with the image-based approach, the average disease severity varied between 16.32% and 88% for Batch-I and between 14.5% and 97.2% for Batch-II (Fig. 3). Likewise, through visual observation, the average disease severity ranged from 6.7% to 93.3% for Batch-I and from 9.0% to 98.0% for Batch-II. The correlations in disease severity ratings in those 2 screening batches were computed using common genotypes as checks, which showed values of 0.40 and 0.53 for image and visual assessments, respectively (Supplementary Fig. 3).

The phenotypic data was also binarized as “0” for more tolerant genotypes and “1” for more susceptible genotypes (Fig. 2). After binarization, there were 8 and 10 individuals in the “0” category within Batch-I for the image and visual approaches, respectively. In Batch-II there were 12 individuals in the “0” category for both approaches. The heritability estimates for anthracnose resistance trait in SHB, in Batch-I was about 0.47, whereas for Batch-II, it was 0.5 (Supplementary Fig. 4), indicating a moderate level of heritability for this trait.

### Genome-wide association analysis

A total of 38,379 SNPs, distributed across the 12 haploid blueberry chromosome-scaled scaffolds, were independently tested for association with anthracnose resistance trait employing a GWAS approach (Supplementary Fig. 5). Population structure was assessed using principal component analysis based on the genomic relationship matrix (Supplementary Fig. 6a). The first 5 PCs each explained more than 1.5% of the total genetic variance, with no clear elbow in the scree plot (Supplementary Fig. 6b). These 5 PCs were included as fixed covariates in the GWAS model to control for population stratification.

**Fig. 4.**
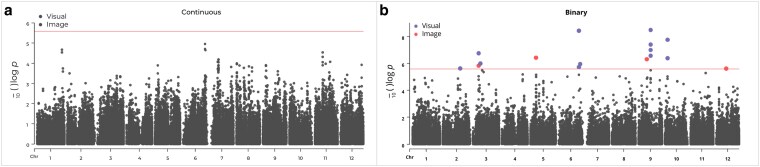
Manhattan plots for anthracnose resistance in the SHB population using 2 distinct phenotyping methods: the image-based approach and the visual approach based on the a) continuous scale b) binarized scale. A linear mixed model with corrections for population structure and cryptic relatedness was used to compute the *P*-values. Adjusted Bonferroni correction, considering a genome-wide significance level of 0.05 (orange line), was used to establish a *P*-value detection threshold for statistical significance.

BLUEs for each genotype, derived from a 1-stage correction of the combined datasets across the 2 screening batches were used as phenotypic inputs for GWAS. In the continuous scale, no significant associations were found despite employing both visual and image approaches for phenotyping (Fig. 4a, Supplementary Fig. 7). With the hypothesis that the unaccounted defoliation discussed earlier may have limited our ability to detect significant associations when the continuous disease severity scale was employed, we further explored the results from the binarized dataset only. Within this binarized dataset, the number and location of significant SNPs varied between visual and image-based approaches (Table 1). The image-based approach revealed associations on chromosomes 3, 5, 9, and 12, whereas the visual approach detected associations on chromosomes 2, 3, 6, 9, and 10 (Fig. 4b, Table 1). QQ-plots showed that the observed *P*-values closely followed the expected null distribution, with no inflation or systematic deviation, indicating minimal population structure or bias in the data and reliable association signals (Supplementary Fig. 8).

**Table 1.** Significant SNPs associated to anthracnose based on binarized scale in SHB population, using 2 different phenotyping approaches: image- and visual-based.

Phenotyping	Chr	Position	*P*-value	MAF	Effect	Score	PVE	Ref/Alt
Image	3	12214430	0.001	0.015	4.21	5.99	3.1	G/C
Image	5	8957403	0.000	0.057	5.19	6.41	4.5	G/C
Image	9	13570208	0.000	0.011	4.97	6.30	5.5	G/A
Image	12	16417571	0.001	0.050	2.22	5.60	3.2	G/T
Visual	2	28697242	0.003	0.140	−13.62	5.61	2.4	G/A
Visual	3	9278591	0.004	0.045	−8.22	6.75	2.3	C/T
Visual	6	33079458	0.007	0.016	−17.03	8.42	2.0	T/C
Visual	9	19827384	0.000	0.056	−11.56	8.47	4.3	C/A
Visual	10	3954703	0.022	0.028	−14.54	7.75	1.5	G/T

The corresponding SNP location, *P*-value, minor allele frequency (MAF), effect, score (i.e. −log_10_[*P*-value]), percentage of variance explained (PVE), and reference (Ref) and alternative (Alt) allele are reported.

### Candidate gene mining

Putative protein-coding genes within a 100 kb window upstream and downstream of significant SNPs obtained in binary scale were retrieved from the reference genome. This window size was selected to account for the rapid LD decay observed in our population (Supplementary Fig. 9), while maintaining consistency with previous studies in blueberry and related crops (Ferrão et al. 2020; Cromie et al. 2025 ). In total, 138 genes were identified across all significant regions and are listed in Supplementary File 4. From these, a subset of genes with annotated functions linked to known defense-related processes was selected for further discussion and is presented in Table 2. Genes encoding for pentatricopeptide repeat-containing protein, E3 ubiquitin protein ligase, and serine/threonine protein kinase were recurrently identified flanking significant SNPs detected either through the image or visual phenotyping approach. The functional relevance of these genes is considered further in the discussion.

**Table 2.** Selected candidate genes related to anthracnose resistance that are within ±100 kb of significant SNPs detected using binary scale.

Gene description	Phenotyping	Chr	Position	Vaccinium gene id
Pentatricopeptide repeat-containing protein	Image	3	12214430	*VaccDscaff4-processed-gene-121.8-mRNA-1*
	Visual	2	28697242	*VaccDscaff2-augustus-gene-286.26-mRNA-1* *VaccDscaff2-processed-gene-286.3-mRNA-1*
		10	3954703	*VaccDscaff20-processed-gene-39.5-mRNA-1*
Serine/threonine-protein	Image	12	16417571	*VaccDscaff22-augustus-gene-164.27-mRNA-1*
	Visual	9	19827384	*VaccDscaff17-processed-gene-198.22-mRNA-1* *VaccDscaff17-augustus-gene-198.26-mRNA-1*
		10	3954703	*VaccDscaff20-snap-gene-39.32-mRNA-1*
E3 ubiquitin-protein	Image	3	12214430	*VaccDscaff4-augustus-gene-121.24-mRNA-1*
	Visual	2	28697242	*VaccDscaff2-snap-gene-287.36-mRNA-1*
		3	9278591	*VaccDscaff4-augustus-gene-92.40-mRNA-1*
		6	33079458	*VaccDscaff11-snap-gene-331.44-mRNA-1* *VaccDscaff11-snap-gene-331.45-mRNA-1*
		10	3954703	*VaccDscaff20-processed-gene-38.9-mRNA-1*
Putative leucine-rich repeat-containing	Visual	3	9278591	*VaccDscaff4-processed-gene-93.2-mRNA-1*
WD domain, G-beta	Image	5	8957403	*VaccDscaff7-augustus-gene-88.22-mRNA-1*
Heat stress transcription factor A-5	Image	3	12214430	*VaccDscaff4-augustus-gene-122.41-mRNA-1*
Cytochrome p450	Visual	2	28697242	*VaccDscaff2-augustus-gene-288.40-mRNA-1* *VaccDscaff2-processed-gene-287.24-mRNA-1* *VaccDscaff2-processed-gene-288.0-mRNA-1*

## Discussion

Developing anthracnose-resistant blueberry cultivars relies on identifying resistance sources and understanding the genetic basis underlying variability in disease severity within the breeding population. Despite its importance, the genetic basis of anthracnose resistance caused by *C. gloeosporioides* in leaves and stems of SHB remains understudied, with most prior studies focusing on anthracnose fruit rot caused by *C. fioriniae* in NHB. Our study evaluated resistance in both leaf and stem tissues, providing a more comprehensive assessment of disease symptoms relevant to Florida's evergreen production system. We conducted GWAS using SHB breeding population to elucidate the genetic and phenotypic variability and identify molecular markers and candidate genes associated with anthracnose resistance in leaves and stem of SHB.

We inoculated detached stem cuttings of genotypes from SHB population with *C. gloeosporioides* in 2 separate screening batches for logistical convenience. While a subset of genotypes was included in both batches, most were unique to each batch. Disease was assessed on day 35 post inoculation with 2 phenotyping approaches: image- and visual-based. In our study, we observed instances of defoliation prior to disease severity rating in certain genotypes exhibiting moderate to severe symptoms of anthracnose, while genotypes with lower symptom levels retained their foliage. This defoliation likely resulted in the disproportionate loss of symptomatic leaves in genotypes with moderate to severe disease, potentially increasing variability in disease severity assessments, particularly among nonresistant genotypes. This unexpected experimental limitation may have impacted our ability to detect significant associations when using continuous disease severity scores and likely affected both visual and image analysis methods. This underscores the logistical challenges of capturing subtle genetic effects using continuous disease severity assessments in a large-scale disease screening.

To address this potential source of error, we decided to binarize the dataset, effectively looking only for genetic signals associated with the most resistant individuals, treating all others as a single comparison group. Binarization emphasized the most pronounced phenotypic contrasts and enable detection of associations that may have been obscured in the continuous dataset. Based on this rationale, we employed a 5th percentile threshold, calculated from each batch's average severity across 3 replicates, to binarize our disease severity data. Individuals with an average disease severity value below the 5th percentile were categorized as 0, indicating minimal symptom development (i.e. resistant), while those above the 5th percentile were categorized as 1 (i.e. susceptible).

Our study revealed a high correlation between image and visual datasets. This approach of image analysis has demonstrated efficacy in evaluating other fruit quality traits, such as wax bloom in blueberry (https://github.com/SFP-team/berrycv-workflow). Moreover, image analysis techniques have proven advantageous in detecting new genetic loci for resistance against pathogens in various plant species, including tomato against *Ralstonia solanacearum*, Arabidopsis against *Sclerotinia sclerotiorum*, and wheat against *Zymoseptoria tritici* (Yates et al. 2019; Barbacci et al. 2020; Meline et al. 2023). Despite being more time-consuming than visual assessment in a study like ours, image analysis remains advantageous, particularly in scenarios when less-trained raters are involved in the scoring of these large populations. In blueberry breeding programs, accurate assessment of anthracnose severity is even more crucial as we observe its complex genetic architecture involving multiple genes (Osorio et al. 2014; Jacobs et al. 2020). So, integrating visual assessment with advanced image analysis techniques offers a promising solution to this challenge. Leveraging these approaches can enhance precision and sensitivity of disease evaluation, minimize biases in traditional assessment methods, and capture more significant associations. In this regard, it is preferable to have false positives for further validation rather than missing potential targets.

We observed a moderate narrow-sense heritability estimate (∼0.5) for anthracnose resistance in SHB which closely aligned with the reported range of 0.63 to 0.73 for anthracnose fruit rot in northern highbush blueberries (Miles and Hancock 2022). This observation underscores the potential for breeding initiatives to effectively exploit genetic variation and enhance resistance against anthracnose.

In our study, no genotype was found to be completely resistant to anthracnose; however, tolerant genotypes with relatively much less disease severity were observed during both screening batches. Five cultivars evaluated in our study: Flicker, Springhigh, Kestrel, Farthing, and Emerald; were also included in a previous anthracnose evaluation by Phillips et al. (2018a). In their study, Flicker was identified as the most susceptible cultivar, while Springhigh was classified as resistant and Kestrel, Farthing, and Emerald exhibited variable levels of susceptibility. In our study, all 5 cultivars showed moderate to high anthracnose severity, with Springhigh consistently exhibiting high symptom levels across both screening batches. This discrepancy likely stems from differences in disease assessment methodology. Phillips et al. (2018a) quantified anthracnose susceptibility based solely on stem lesion counts and stem lesion length, whereas our approach incorporated a more comprehensive evaluation by estimating the percentage of necrotic tissue across entire stem cuttings and associated leaves. This broader assessment captures the full spectrum of symptom expression under natural field conditions, including foliar necrosis that may be overlooked in stem-focused evaluations.

The varying degrees of response to anthracnose observed among different individuals underscores the presence of genetic variability within our population. Our GWAS analysis revealed the presence of significant associations across several genomic regions spanning different chromosomes. The presence of multiple minor additive genes contributing to a small portion of the phenotypic variance in blueberries is consistent to a complex and quantitative nature of anthracnose resistance as observed in other species like pepper, tea, cassava, sorghum, and beans (Oh et al. 2003; Boonchanawiwat et al. 2016; Perseguini et al. 2016; Mishra et al. 2017; Prom et al. 2019; Ro et al. 2023). While our study primarily focuses on identifying genetic factors associated with resistance in leaf and stem tissues, potentially differing from those relevant in fruits, it is important to acknowledge limitations inherent in our phenotyping strategy. The artificial inoculation method used, featuring high inoculum density (Jacobs et al. 2020) and close tissue proximity may have overwhelmed subtle resistance mechanisms, potentially masking genetic variation relevant under natural field conditions. Additionally, although the detached stem assay offers advantages such as rapid, high-throughput screening and preservation of germplasm (Miller-Butler et al. 2018), it lacks whole-plant physiological responses and may introduce variability due to slight differences in stem maturity or postexcision effects (Pettitt et al. 2011). These factors could contribute to the moderate correlation observed between resistant and susceptible checks across the screening batches. Thus, while effective for larger-scale screening, detached assays should be complemented with whole-plant evaluations in advanced breeding stages to ensure accurate assessment of durable, field-relevant resistance.

Despite these phenotyping limitations, our genomic analysis identified significant SNPs on chromosome 9, consistent with findings from Jacobs et al. (2023). However, the specific SNP locations differed between the 2 studies. This discrepancy may reflect the complexity of host defense responses in this region, which are likely governed by polygenic networks and genes involved in quantitative or broad-spectrum resistance rather than major-effect loci. Such complexity can result in variable association signals depending on the phenotyping approach, environmental context, or genetic background of the population studied.

Based on the significant genomic regions detected via GWAS, we highlight candidate genes potentially involved in defense-related pathways. While this study does not directly elucidate the molecular mechanisms and causal mutations underlying resistance, it provides potential candidates that could be targeted for further functional validation. The candidate genes identified span diverse functional categories, including plant defense regulation, stress signaling, and broader cellular response mechanisms. In the sections below, we reflect on the multifaceted nature of defense mechanisms and discuss genes associated with distinct layers of defense response.

Fundamental to plant defense is the process of pathogen recognition. Among our candidate genes, we identified one containing leucine-rich repeats, a structural feature essential in plant immune receptors involved in detecting pathogen effectors and activating defense responses against pathogens such as C*olletotrichum (Padmanabhan et al. 2009; Li et al. 2013; Jang et al. 2020; da Silva Dambroz et al. 2023)*.

A second critical layer of defense involves the detoxification of reactive oxygen species (ROS) and reinforcement of pathogen entry barriers. During stress or infection, ROS can accumulate in mitochondria, chloroplasts, and the apoplast, leading to tissue damage (Kaur et al. 2022). Among our candidates, heat stress transcription factor A-5 plays a key role in regulating cell death under oxidative stress (Baniwal et al. 2007). Similarly, pentatricopeptide repeat-containing proteins contribute to mitochondrial ROS homeostasis (Geddy and Brown 2007; Laluk et al. 2011) and have been linked to *Colletotrichum* resistance in pepper (Ro et al. 2023) and sorghum (Prom et al. 2019).

Signaling cascades represent a critical layer of defense, coordinating responses to pathogen attack and enhancing immunity. Our candidate genes include serine/threonine protein kinases and phosphatases, which have been associated with resistance to *C. gloeosporioides* in walnut (An and Yang 2014), *C. lindemuthianum* in bean (Chen et al. 2017), and other pathogens in various plant species (Martin et al. 1993; Song et al. 1995; Salmeron et al. 1996; Warren et al. 1998). We also identified a gene encoding a WD domain, G-beta repeat-containing protein (WD40 family), which may support protein–protein interactions and defense-related signal transduction (Villanueva et al. 2016).

We also detected genes involved in ubiquitin-mediated defense pathways, including E3 ubiquitin ligases and RING-type E3 ubiquitin transferases. These gene families are well established in mediating innate immune responses (You et al. 2016), modulating phytohormone signaling, and conferring broad-spectrum resistance (Kelley 2018). Members of this family have been linked to resistance against *Colletotrichum* species in pepper (Ro et al. 2023) and against *Puccinia* species in wheat (Shahinnia et al. 2022). In addition, we identified genes involved in hormonal signaling, including clusters of cytochrome P450s, a family associated with *C. gloeosporioides* resistance in pepper (Oh et al. 1999; Ko et al. 2005).

Although still speculative, our findings supported by functional annotations and prior literature, particularly against *Colletotrichum* species, strengthen the hypothesis that the identified candidate genes contribute to resistance mechanisms. We acknowledge that additional nearby or uncharacterized genes may also be involved, and further investigation is required to confirm their functional relevance.

The observed phenotypic variability among genotypes, coupled with the detection of multiple loci with minor additive effects, supports the polygenic and quantitative nature of anthracnose resistance in SHB. While the low phenotypic variance explained by individual markers currently limits their utility for marker-assisted selection (MAS), these associations lay important groundwork for predictive breeding. To support these efforts, the high-resolution phenotyping protocol developed in this study, designed to capture the full spectrum of disease symptoms in leaves and stems under field-relevant conditions, can be integrated into breeding pipelines to enhance the accuracy of resistance screening and genomic model training. As mapping resolution improves and additional functional variants are validated, MAS could become feasible for specific loci. In the meantime, the results strongly support the potential of genomic selection, which is better suited for traits controlled by numerous small-effect loci.

As a next step, we will validate the expression of selected candidate genes with strong functional relevance to better understand the biological mechanisms underlying anthracnose resistance. In parallel, we will evaluate the predictive power of genome-wide markers using genomic selection models. Together, these complementary approaches will deepen insight into resistance pathways and aid in identifying marker sets with practical utility in predictive breeding, ultimately guiding the development of cultivars with improved and durable anthracnose resistance.

## Conclusion

This study aimed to elucidate the genetic basis of anthracnose resistance on blueberry leaves and stems. We established a screening protocol to assess anthracnose severity and applied it across 2 screening batches using advanced selections from the UF Blueberry Breeding Program. Then, through GWAS, we identified several genomic regions associated with anthracnose resistance against *C. gloeosporioides* using both computer vision phenomics and human vision. Our findings highlight the polygenic nature of this trait, with resistance governed by multiple minor-effect loci. Candidate genes identified within associated regions suggest involvement in diverse plant defense pathways, providing targets for future functional validation. The screening protocol employed in this study can be integrated into the breeding pipeline to screen further selections against anthracnose, and the identified markers can serve as valuable genomic resources for expediting the development of anthracnose-resistant blueberry varieties.

## Supplementary Material

jkaf187_Supplementary_Data

## Data Availability

The genotypic data used in the analysis are available in Supplementary File 1. Supplementary File 2 contains the phenotypic data used in the analyses. Supplementary Files 3 and 4 contains the pedigree matrix and genomic relationship matrix among individuals in the studied population respectively. Supplementary File 5 contains the functional annotation of candidate genes located in genomic regions of significant SNPs. Supplemental material available at G3 online.
