# Supplementary material for: Genome-wide association study reveals candidate loci for resistance to anthracnose in blueberry
Source: G3 (Bethesda). 2025 Sep 8;15(11):jkaf187. doi: 10.1093/g3journal/jkaf187 (PMC12610935; doi:10.1093/g3journal/jkaf187)
Supplement: jkaf187_Supplementary_Data [file jkaf187_supplementary_data.zip › Supplemental_Figures_G3-2025-406108.docx]

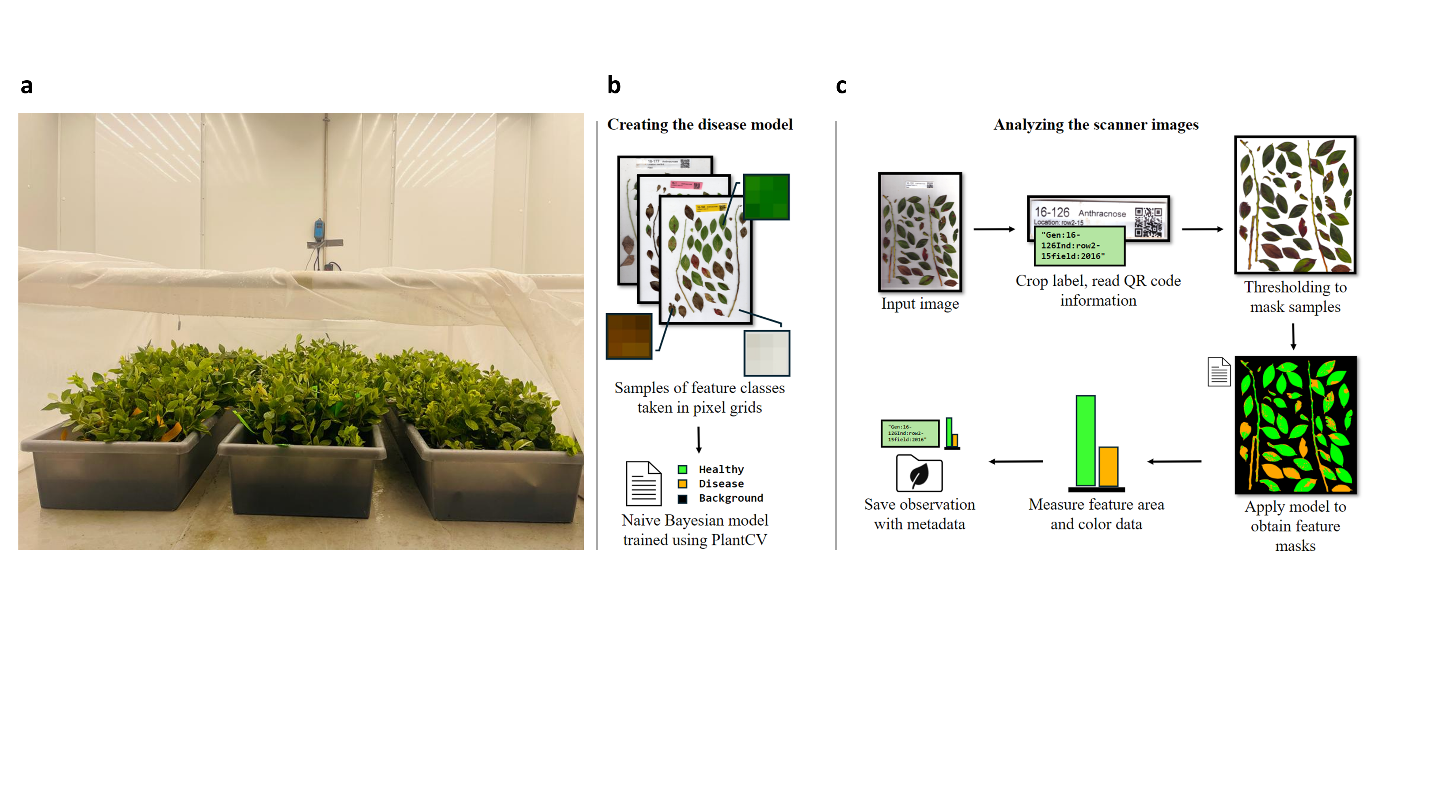


**Fig S1**: Disease assessment of southern highbush blueberry genotypes to anthracnose (a) Incubation of southern highbush blueberry genotypes post inoculation with *Colletotrichum gloeosporioides* inside a plastic-tent housed in growth chamber. Each grey-colored trough represents one out of three replicates for one screening batch. (b) Generation of a disease model within the image analysis framework for assessment of SHB genotypes against anthracnose. (c) Evaluation of acquired images employing the disease model integrated into the image analysis pipeline.


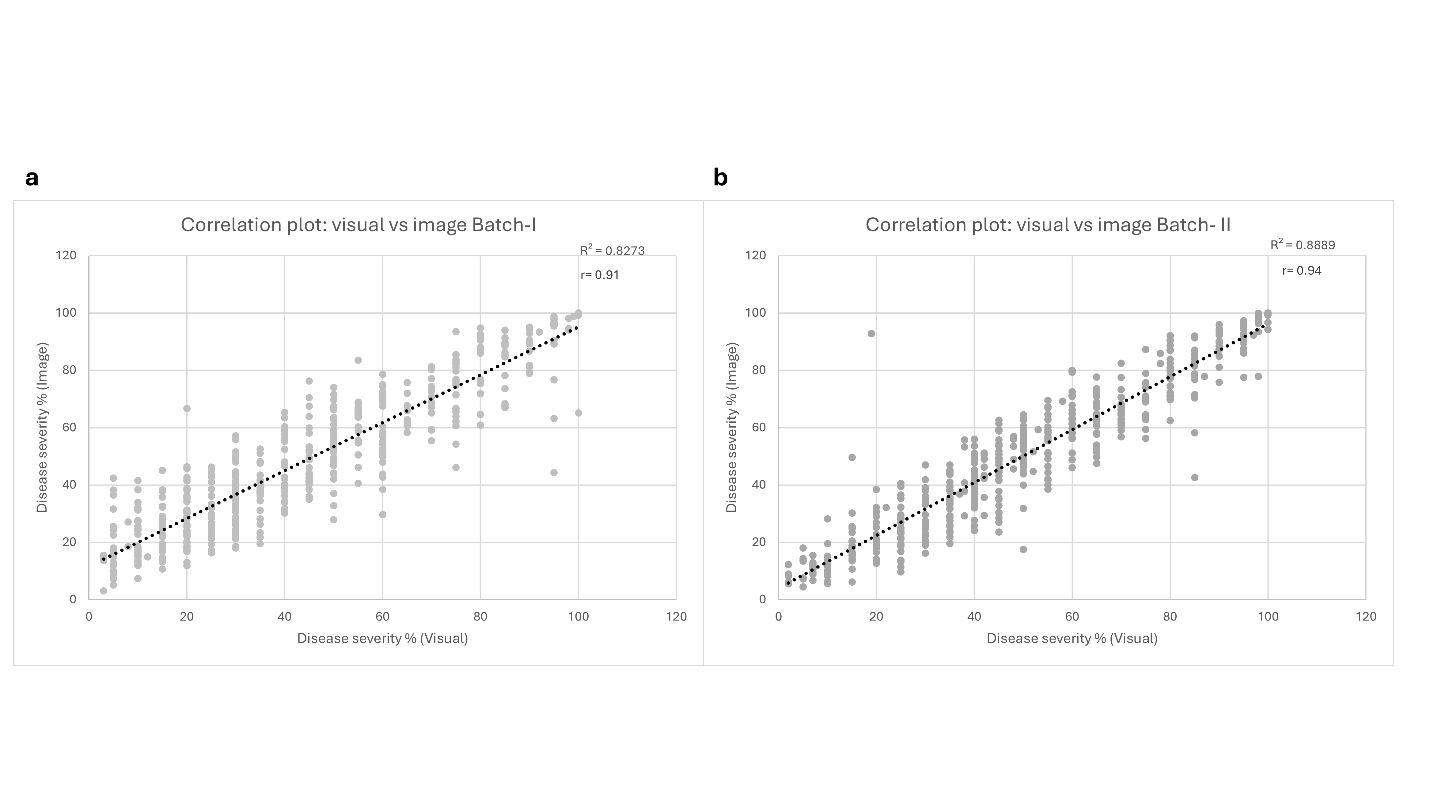


**Fig S2**: Scatterplot showing the correlation between two phenotyping approaches: image- and visual-based within two screening batches: (A) Batch-I and (B) Batch-II in a continuous scale.


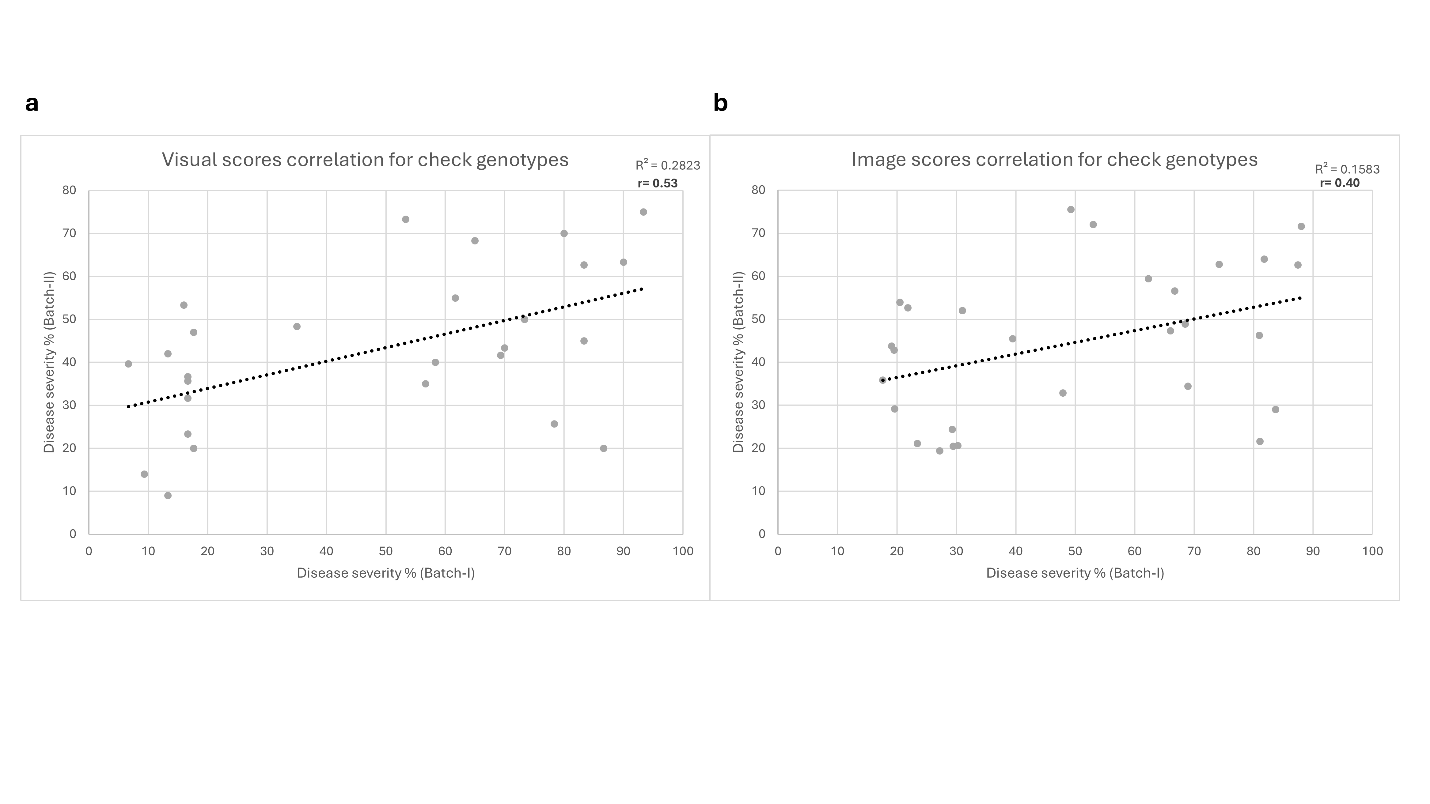


**Fig S3**: Correlation of disease severity scores for overlapping check genotypes across two screening batches, Batch-I and Batch-II, evaluated using two phenotyping approaches: a) visual-based b) image-based

.
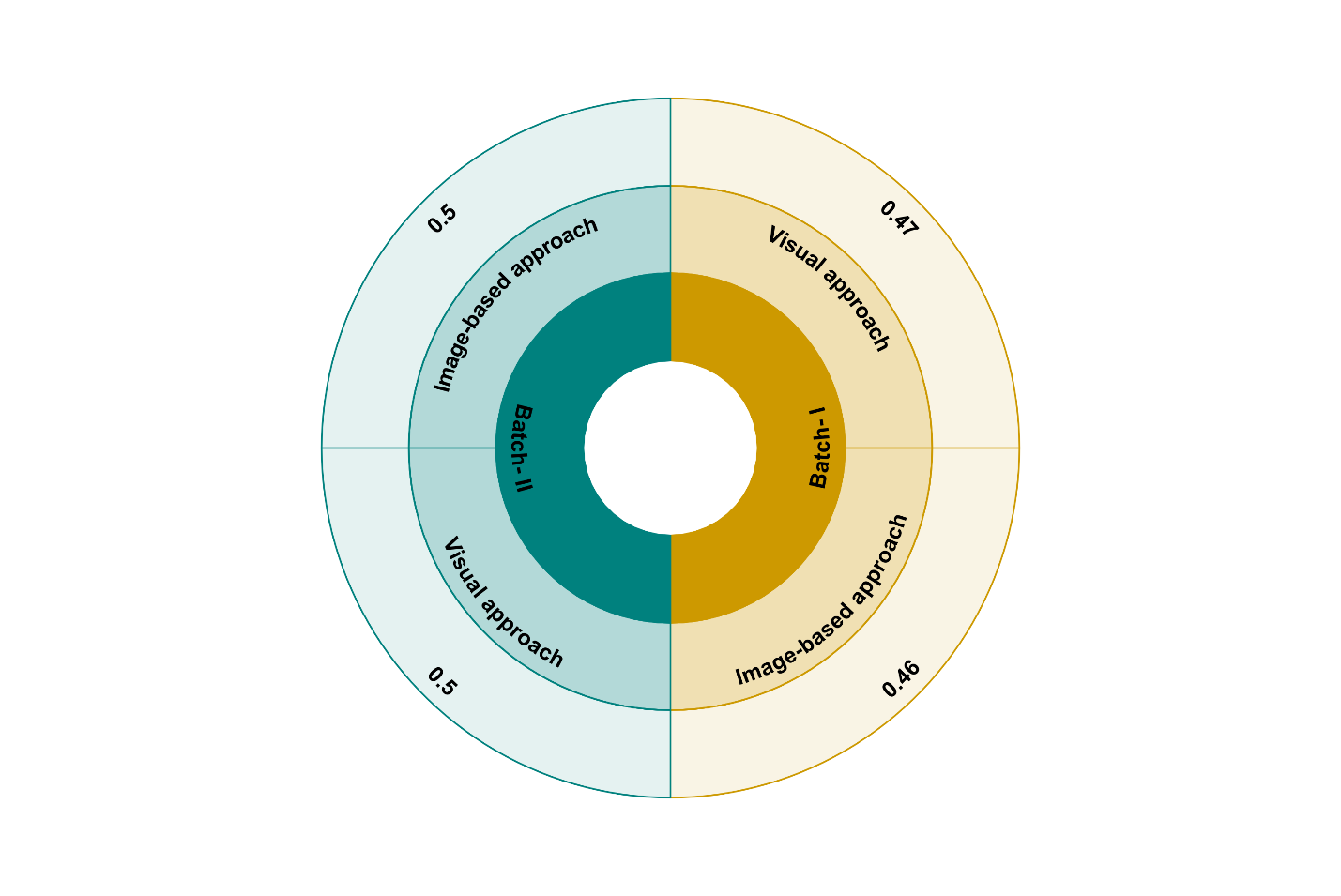


**Fig S4**: Narrow-sense heritability estimates for anthracnose resistance in SHB phenotyping population across two screening batches: Batch-I and Batch-II with two different phenotyping approaches


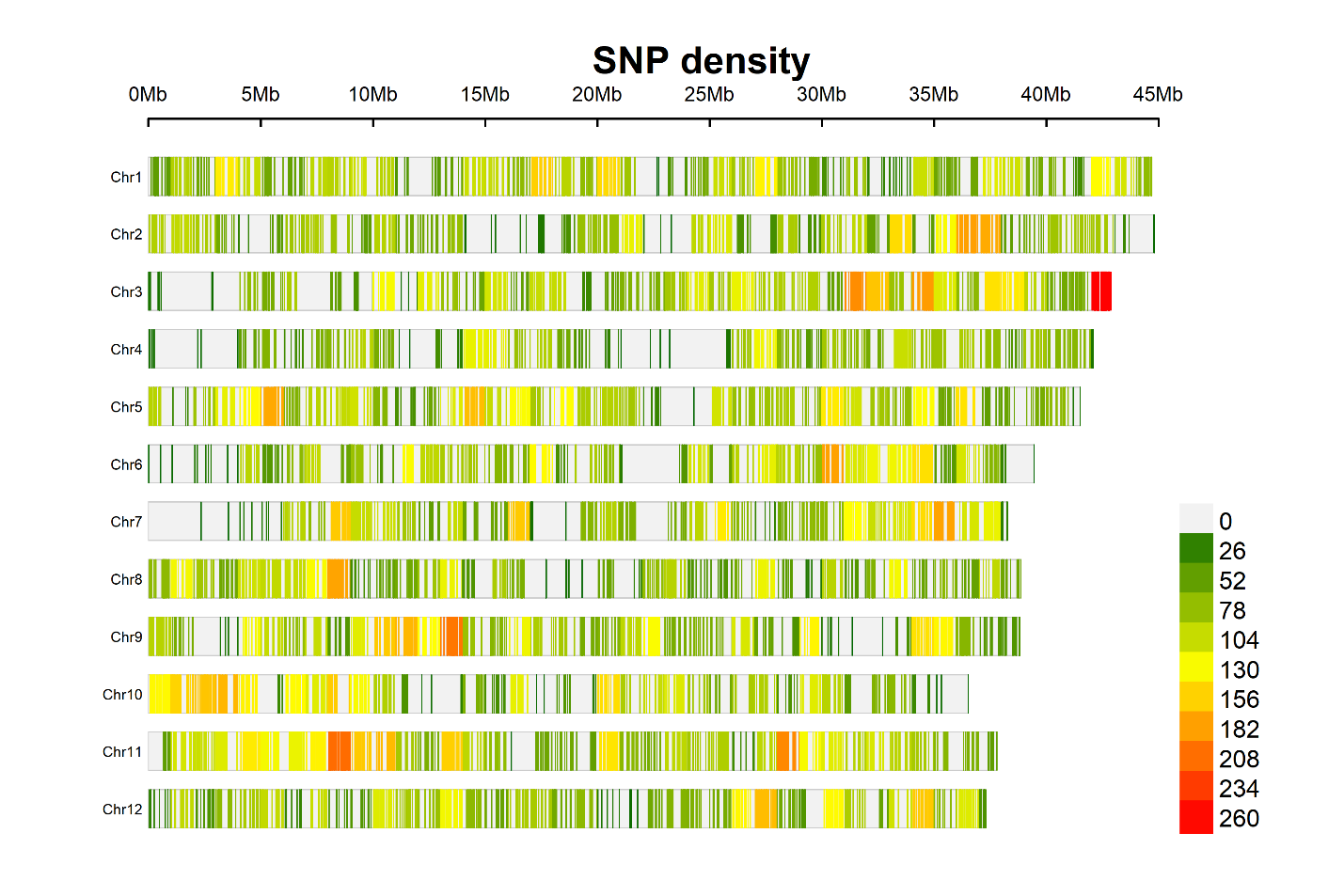


**Fig S5**: Distribution of 38,379 filtered single nucleotide polymorphisms (SNPs) in 1 Mb window size across the 12 blueberry chromosomes. The x-axis represents the distance in base pairs.


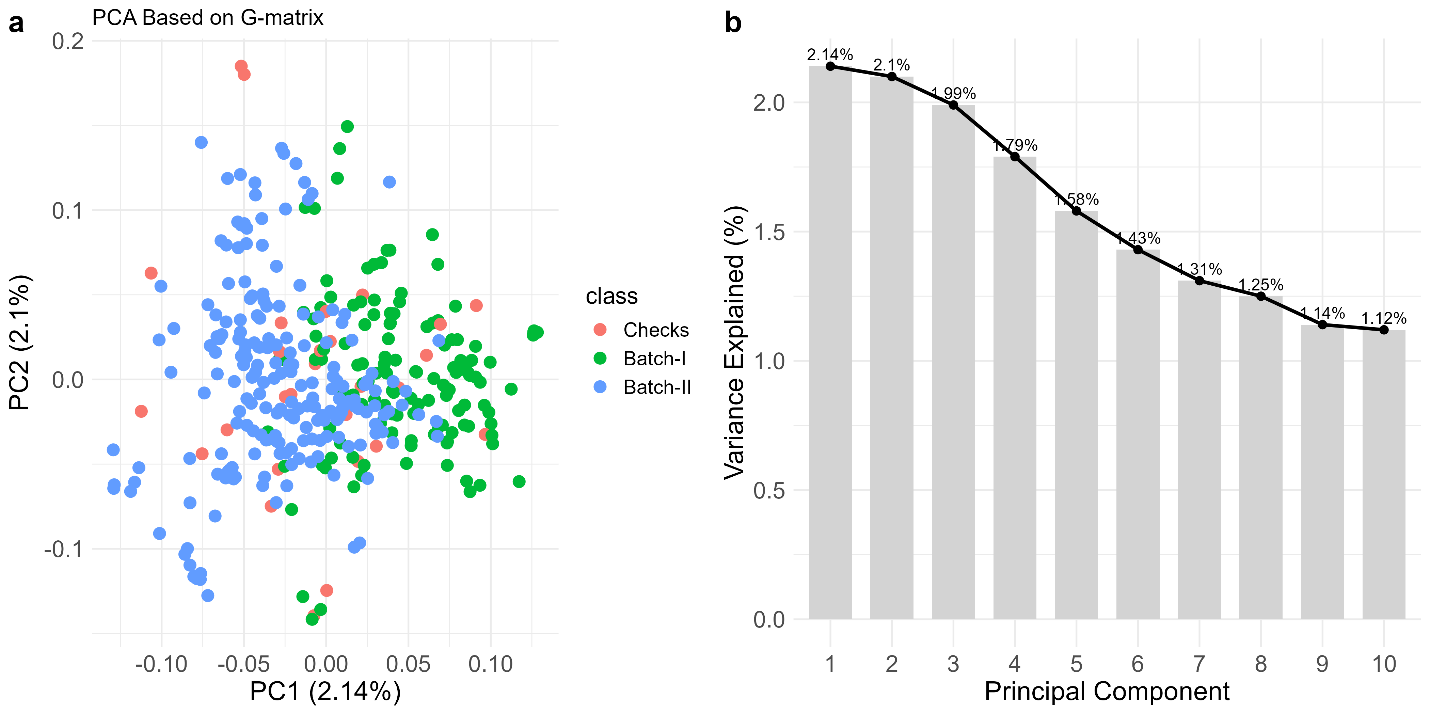


**Fig S6**: a) Genomic PCA based on the SNP-derived genomic relationship matrix for 355 southern highbush blueberry genotypes screened across two batches (Batch-I and Batch-II). Each point represents an individual in the hyperspace defined by the eigenvectors of the first and second principal components. b) Scree plot showing the contribution of the first ten principal components to the underlying population structure.


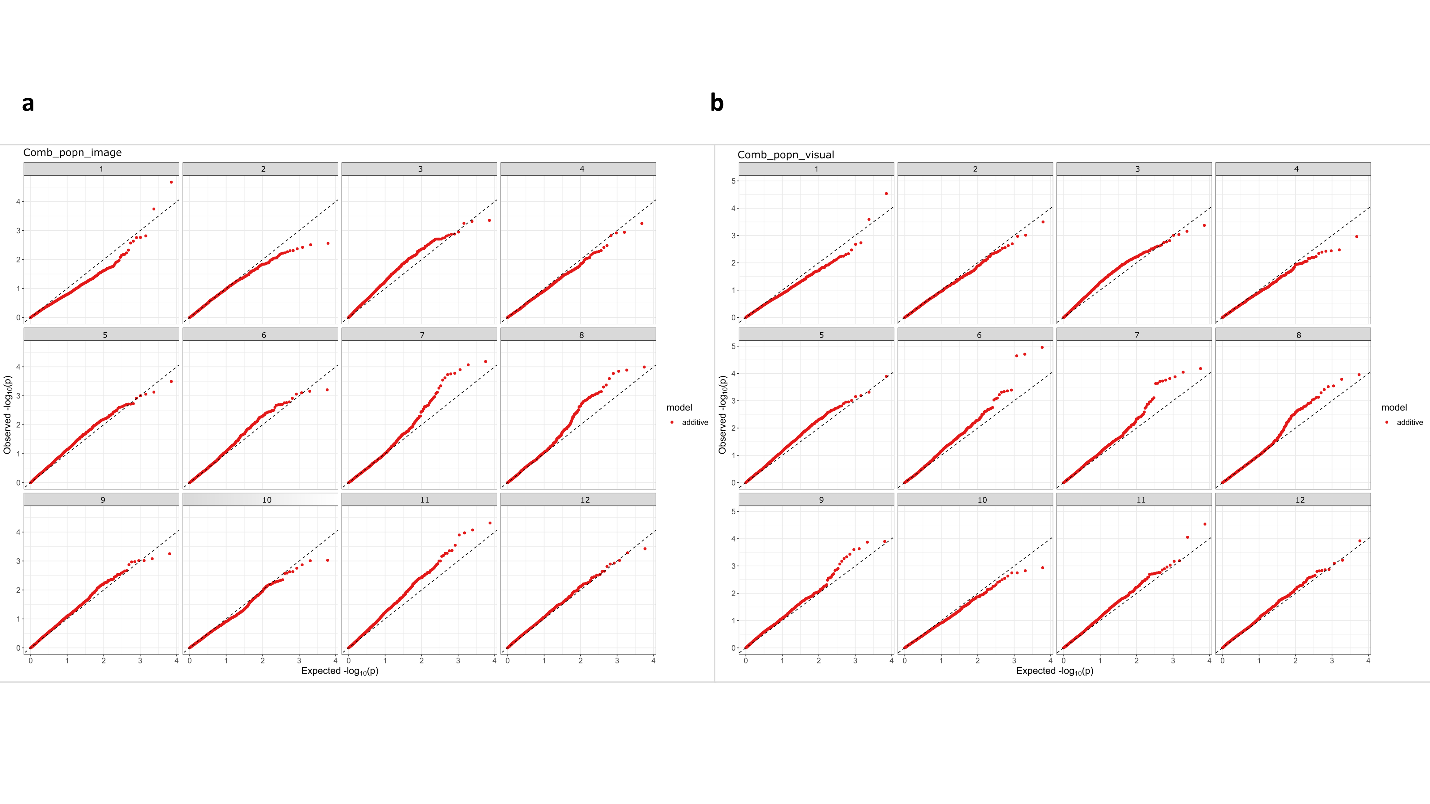


**Fig S7**: Quantile-quantile (Q-Q) plots of GWAS results for anthracnose resistance in SHB genotypes, based on the continuous scale using two different phenotyping approaches a) using image-based approach b) using visual approach. The observed association significance is plotted against the expected association significance for each SNP.


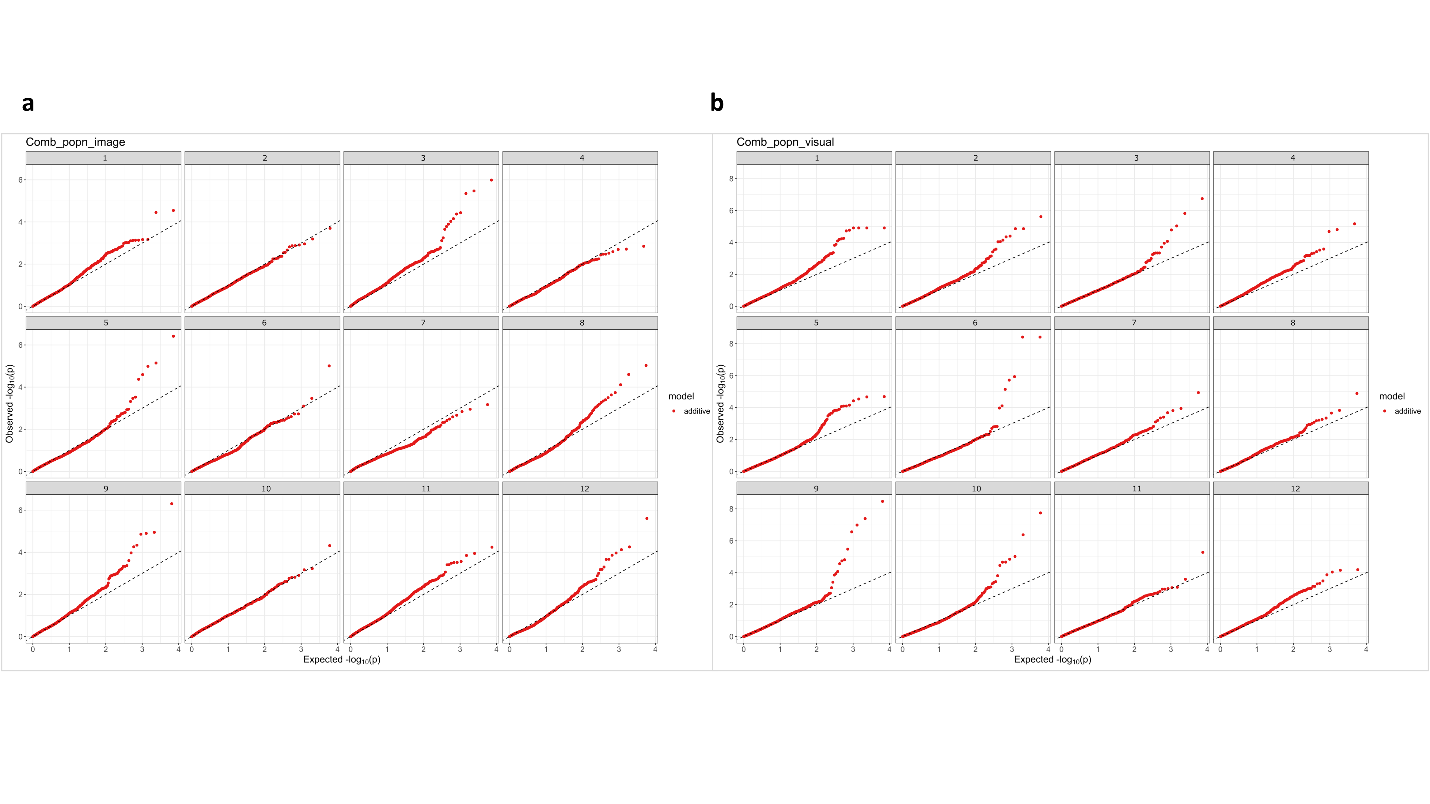


**Fig S8**: Quantile-quantile (Q-Q) plots of GWAS results for anthracnose resistance trait in SHB genotypes, based on the binarized scale using two different phenotyping approaches a) using image-based approach b) using visual approach. The observed association significance is plotted against the expected association significance for each SNP


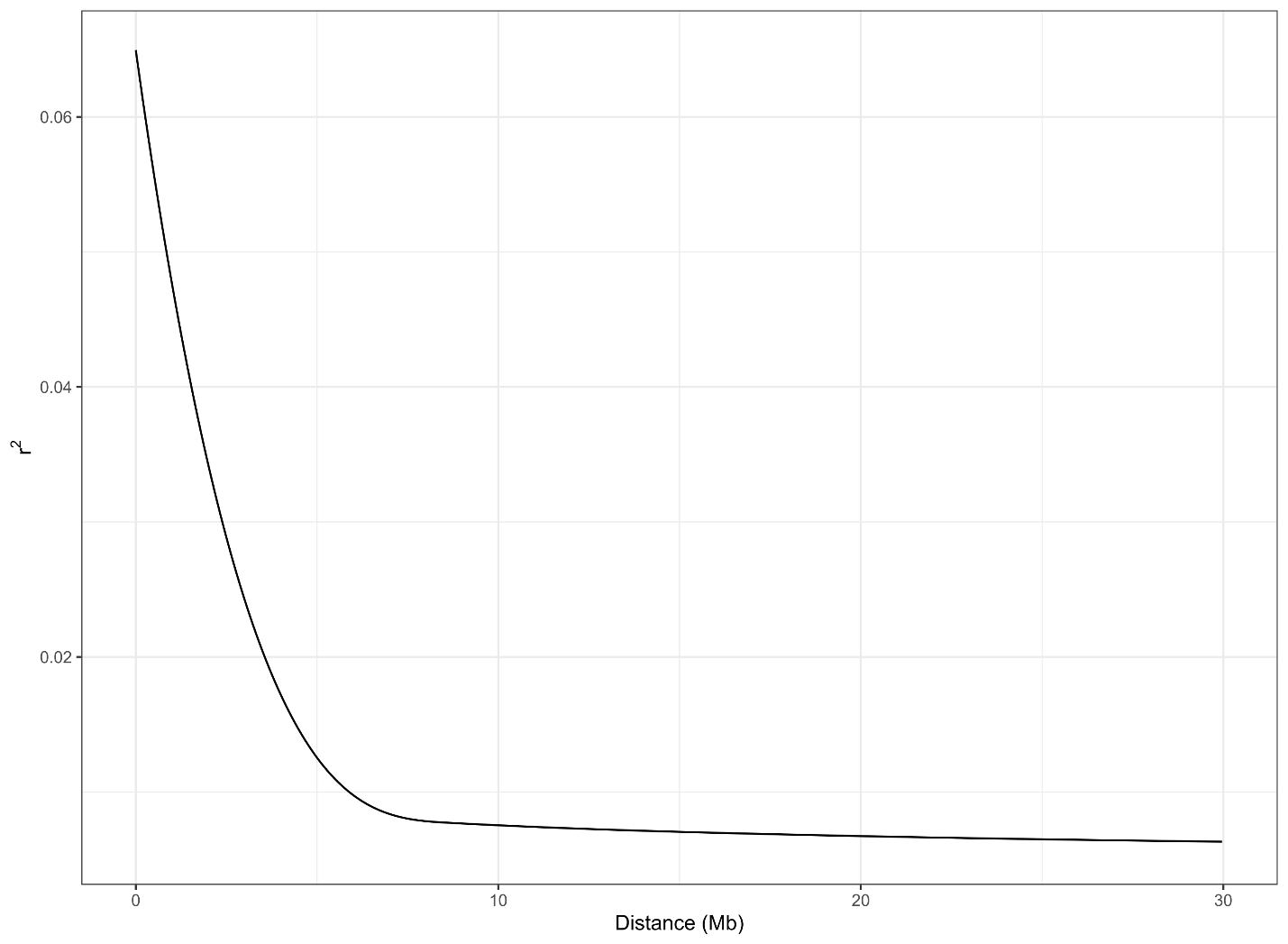


**Fig S9**: Linkage disequilibrium decay in the studied SHB population, calculated using up to 1,000 loci per chromosome. Physical distances between SNPs were based on the *Vaccinium corymbosum* ‘Draper’ reference genome.
